# Cerebellar Degeneration Impairs Strategy Discovery but Not Strategy Recall

**DOI:** 10.1007/s12311-022-01500-6

**Published:** 2022-12-05

**Authors:** Jonathan S. Tsay, Lauren Schuck, Richard B. Ivry

**Affiliations:** 1 Department of Psychology, University of California, Berkeley, CA, USA; 2 Helen Wills Neuroscience Institute, University of California, Berkeley, CA, USA

**Keywords:** Motor learning, Visuomotor adaptation, Decision making, Cerebellar degeneration

## Abstract

The cerebellum is recognized to play a critical role in the automatic and implicit process by which movement errors are used to keep the sensorimotor system precisely calibrated. However, its role in other learning processes frequently engaged during sensorimotor adaptation tasks remains unclear. In the present study, we tested the performance of individuals with cerebellar degeneration on a variant of a visuomotor adaptation task in which learning requires the use of strategic re-aiming, a process that can nullify movement errors in a rapid and volitional manner. Our design allowed us to assess two components of this learning process, the discovery of an appropriate strategy and the recall of a learned strategy. Participants were exposed to a 60° visuomotor rotation twice, with the initial exposure block assessing strategy discovery and the re-exposure block assessing strategy recall. Compared to age-matched controls, individuals with cerebellar degeneration were slower to derive an appropriate aiming strategy in the initial Discovery block but exhibited similar recall of the aiming strategy during the Recall block. This dissociation underscores the multi-faceted contributions of the cerebellum to sensorimotor learning, highlighting one way in which this subcortical structure facilitates volitional action selection.

## Introduction

Motor adaptation ensures that well-learned movements remain accurate across a broad range of contexts [1, 2]. For example, a boxer fights against fatigue to keep his arms up and maintains a proper defensive posture, while a golfer strategically re-aims her swing to counteract a strong crosswind.

Adaptation does not rely on a unitary process, but instead engages multiple learning mechanisms. A basic dichotomy delineates these processes into two general categories [1–9], those that entail volitional strategic changes associated with action selection [10] and those that are implicit, operating in an automatic way to ensure the sensorimotor system is properly calibrated [11–14]. To illustrate this dichotomy, Stephen Curry can volitionally adjust his shooting angle to avoid an opponent seeking to block his shot, while subconsciously and automatically fine-tune the effort required to maintain a stable shooting angle in response to muscular fatigue.

It is clear from previous studies that the cerebellum plays a key role in implicitly recalibrating the sensorimotor system in response to force field and visuomotor perturbations [9, 15–20]. Implicit recalibration is severely attenuated in patients with cerebellar degeneration compared to their age-matched controls [11, 15, 17, 20–23], and cerebellar lesions in non-human animals produce impairments in a wide range of adaptation tasks [24–26]. Similarly, physiological correlates of implicit recalibration are prominent in the cerebellum [27–33].

More recently, researchers have begun to examine the contribution of the cerebellum to strategic processes engaged in motor adaptation. This work has generally involved variants of a visuomotor adaptation task in which participants move in a virtual reality setup where the vision of the arm is occluded, and feedback is limited to a cursor. Adaptation processes are engaged by manipulating the relationship between the arm and cursor, usually by imposing a rotational transformation. While the resultant error will automatically engage implicit recalibration, participants are also likely to strategically adjust their aim to offset the perturbation, especially if the perturbation is large (e.g., aim in a clockwise direction in response to a 60° counterclockwise rotation). Individuals with cerebellar degeneration (CD) are able to employ the appropriate re-aiming strategy if provided with verbal instructions and/or visual cues [34, 35]. In contrast, CD participants exhibit a marked deficit in re-aiming when required to discover the appropriate strategy [36].

Inspired by this dissociation across studies, we hypothesized that the cerebellum may be selectively involved in the *discovery* of a re-aiming strategy, rather than the *recall* of a known strategy. To test this, we recruited participants with CD and age-matched controls to participate in a visuomotor adaptation task, one designed to isolate these components of strategic re-aiming. Critically, the participants were exposed to the perturbation twice, with the initial exposure block assessing strategy discovery and the re-exposure block assessing strategy recall. A deficit in strategy discovery in the CD group will manifest as less effective re-aiming during the initial exposure block compared to their age-matched controls, whereas a deficit in strategy recall will manifest as less effective re-aiming during the re-exposure block. Taken together, these two probes will specify how the cerebellum may contribute to strategic use during sensorimotor learning.

## Materials and Methods

### Ethics Statement

All participants gave written informed consent in accordance with policies approved by the UC Berkeley Institutional Review Board. Participation in the study was in exchange for monetary compensation.

## Participants

We recruited 16 individuals with a medical diagnosis of cerebellar degeneration (CD) from our clinical database. The database is composed of individuals from around the country who responded to online advertisements either posted on the National Ataxia Foundation website or distributed by leaders of local support groups. For those who agreed to participate, we conducted an online video interview to obtain a medical history, a neurological assessment of CD symptoms using the Scale for Assessment and Rating of Ataxia (SARA; [37]), and an evaluation of general cognitive status using the Montreal Cognitive Assessment (MoCA; [38, 39]). Table 1 provides a summary of group demographics and Table 2 provides this information for each participant.

The sample size was based on an analysis of the data from a published study comparing strategic re-aiming in CD and age-matched controls [36]. In that study, groups of 10 CD and control participants yielded a large effect size of 3.75 and a power of 0.99. We opted to recruit 16 individuals for our groups as a conservative and feasible sample size.

Inclusion in the CD group was based on genetic confirmation of spinocerebellar atrophy (SCA) or a clinical diagnosis of ataxia. Nine of the 16 individuals had an identified subtype (SCA1: 1; SCA2: 2; SCA3: 5; SCA6: 1). One individual in the CD group was classified as SAOA (sporadic adult-onset ataxia). Six individuals did not have genetic testing (i.e., unknown etiology) but had MRI confirmation of cerebellar degeneration. Based on the medical history component of the online interview, we excluded candidate participants who self-reported ataxia but neither had MRI confirmation of cerebellar degeneration or genetic confirmation of SCA.

The SARA and MoCA were modified for online administration [40]. For the SARA, two items were scored based on self-reports due to concerns about safety during remote evaluation (Gait and Stance). For the MoCA, we eliminated the Alternating Trail Making Task in the online version because it requires a paper copy. The scores were rescaled to the standard score range (0–30). As shown in Table 1, the CD group exhibited moderately severe cerebellar ataxia (a SARA score greater than 14) [37]. Five of the CD participants exhibited mild cognitive impairment (a MoCA score less than 26) [38]. We opted to include these participants given our general interest in the contribution of the cerebellum to cognition, and here in this study, with a specific focus on testing the impact of CD on strategic re-aiming.

We recruited a sample of 16 matched controls, all of whom do not have a history of neurological disorders. The CD and Control groups did not show significant differences in terms of sex, handedness, age (*t*(30) = 0.5, *p* = 0.60,*D* = 0.2), years of education (*t*(30) = 0.8, *p* = 0.50,*D* = 0.3), and MoCA scores (*t*9(30) = 1.6, *p* = 0.10,*D* = 0.6).

## Apparatus and General Procedures

Participants used their own laptop or desktop computer to access a customized webpage that hosted the experiment [41]. To provide instructions and ensure task compliance, an experimenter was present through a video link during the 1-h experimental session. Participants used their computer trackpad or mouse to perform the reaching task (sampling rate typically ~ 60 Hz). The size and position of stimuli were scaled based on each participant’s screen size. For ease of exposition, the stimuli parameters reported below are for a typical monitor size of 13″ with a screen resolution of 1366 × 768 pixels [42].

Reaching movements were performed by using the computer trackpad or mouse to move the cursor across the monitor. Each trial involved a planar movement from the center of the workspace to a visual target. The center position was indicated by a white circle and the target location was indicated by a blue circle (both 0.5 cm in diameter). On the typical monitor, the radial distance from the start location to the target was 6 cm. The target appeared at one of two locations on an invisible virtual circle (60° = upper right quadrant; 210° = lower left quadrant). The movement involved some combination of joint rotations about the arm, wrist, and/or finger depending on whether the trackpad or mouse was used. In our prior validation work using this online interface and procedure, the exact movement and the exact device used did not impact measures of performance or learning on visuomotor adaptation tasks [41]. We note that, unlike our laboratory-based setup in which we occlude vision of the reaching hand, this was not possible with our online testing protocol. However, we have found that measures of implicit and explicit adaptation are similar between in-person and online settings. Moreover, based on informal observation, participants remain focused on the screen during the experiment (to see the target and how well they are doing). The vision of the hand would be limited to the periphery.

To initiate each trial, the participant moved their cursor to the circle positioned at the center of the screen, the start location. The cursor was only visible when the white dot was within 2 cm of the start location. This minimized adaptation that might occur in response to feedback from the cursor during the return movement to the start location. Once the participant maintained their position in the starting circle for 500 ms, a blue circle would appear at one of the two target locations. The participant was instructed to reach after they heard the go-cue (i.e., an auditory “beep”), attempting to make the cursor land in the target. To minimize demands on amplitude control and/or movement termination, they were instructed to reach past the target.

We imposed a delay, randomly selected from rectangular distribution ranging from 1100–1300 ms between the target appearance and the auditory go-cue. By imposing a delay between target presentation and movement initiation, we sought to nullify any group differences in the time required for movement preparation and, as such, eliminate potential speed-accuracy differences. Participants heard the message “Wait for the tone!” if they initiated a movement prior to the go-cue and heard the message “Move earlier!” if they initiated a movement 800 ms after the tone.

The visual feedback cursor during the center-out movement could take one of three forms: congruent feedback, rotated feedback, and no feedback. For the congruent and rotated conditions, the cursor appeared at a single location (“endpoint feedback”). During congruent feedback trials, the angular position of the visual feedback was aligned with the direction of the hand movement. Thus, if the participant’s movement sliced through the target, the feedback appeared at the target location. During rotated feedback trials, the angular position of the cursor was rotated 60° relative to the position of the participant’s hand. Thus, if the participant’s movement sliced through the target, the position of the feedback cursor was shifted 60° along an invisible circle with a circumference equal to the target amplitude. The direction of this offset was either clockwise or counterclockwise (counterbalanced across participants and targets). During no-feedback trials, the cursor was extinguished as soon as the hand left the start circle and remained off for the entire movement.

Critically, when visual feedback was provided (congruent or rotated), we imposed an 800 ms delay between the time at which the amplitude of the movement reached the target distance and the onset of the visual feedback. By delaying the visual feedback, we sought to eliminate the contribution of implicit processes that occur during motor adaptation, and as such, isolate the contribution of strategic re-aiming [43–46]. The feedback cursor remained visible for 500 ms.

## Experimental Design

The experiment consisted of three phases. First, participants watched a video that introduced the various manipulations in the task, emphasizing that the primary goal was to hit the target with their visual cursor. The video also highlighted the use of a delayed go-cue and delayed feedback. Second, to become familiar with the web-based reaching environment as well as the congruent and no-feedback conditions, the participants completed 15 practice trials. Third, participants completed the main task, which entailed six blocks (80 movement cycles × 2 targets = 160 trials total): a baseline veridical feedback block (5 cycles), a rotated contingent feedback block (i.e., “Discovery” block; 30 cycles), an initial no-feedback aftereffect block (5 cycles), veridical feedback block (5 cycles), rotated contingent feedback block (i.e., “Recall” block; 30 cycles), and a second no-feedback aftereffect block (5 cycles).

Before the start of each veridical feedback block, participants were provided the following instructions: “Please move your white cursor directly to the blue target immediately after the tone.” Before the start of each rotated contingent feedback block, participants were provided the following instructions: “Your white cursor will be offset from where you move. Hit the blue target with your white cursor.” Before the start of each no-feedback aftereffect block, participants were provided the following instructions: “Your white cursor will be hidden and no longer offset from where you moved. Please move directly and immediately to the blue target after the tone.”

## Data Analysis

All data and statistical analyses were performed in *R*. The primary dependent variable was the endpoint hand angle on each trial, defined as the angle of the hand relative to when the movement amplitude reached a 6-cm radial distance from the start position. To aid visualization, the data were collapsed across the two movements in a cycle (one per target). Data points were considered outliers when the hand angle deviated from a 5-trial trendline by more than 3 standard deviations. This resulted in the exclusion of 3.2 ± 1.9% (mean ± SD) of the data points from the control group and 2.7 ± 1.6% of the data points in the CD group.

We compared hand angle between groups in three a priori-defined epochs [47]: early adaptation, late adaptation, and aftereffect. Early adaptation was defined as the initial ten movement cycles after the rotation was introduced (Discovery block: cycles 7–16; Recall block: cycles 47–56). Late adaptation was defined as the final ten movement cycles of the rotation blocks (Discovery block: cycles 31–40; Recall block: cycles 66–75). After effect was defined as all movement cycles without visual feedback after the rotation was removed (following the Discovery block: cycles 41–45; following the Recall block: cycles 76–80).

Reaction time was defined as the time between the go-cue and the start of the movement, with the latter operationalized as the time at which the hand movement exceeded 1 cm. Movement time was defined as the time from the initial movement to the time when the cursor reached 6 cm. Reaction time (control: 499.4 ms (182.4, 747.5); CD: 469.8 ms (− 53.3, 1043.6); *t*(29) = 0.7, *p* = 0.5,*D* = 0.2) and movement time (control: 499.4 ms (115.5, 1167.8); CD: 410.5 ms (120.5, 1005.5); *t*(29) = 0.7, *p* = 0.5,*D* = 0.3) did not significantly differ between groups.

We employed *F*-tests with the Satterthwaite method to evaluate whether the coefficients (i.e., beta values) obtained from the linear mixed effects model were statistically significant (*R* functions: lmer, lmerTest, ANOVA). Pairwise post hoc two-tailed *t*-tests (or Wilcoxon sign-ranked test when parametric assumptions were violated) were employed to evaluate whether hand angle and kinematic measures differed between groups. *P*-values were adjusted for multiple comparisons using the Tukey method. The degrees of freedom were also adjusted when the variances between groups were not equal. Standard effect size measures are also provided (*D* for between-participant comparisons; *D* for within-participant comparisons; $\eta_{p}^{2}$ for between-subjects ANOVA) [48].

## Results

**Fig. 1:**
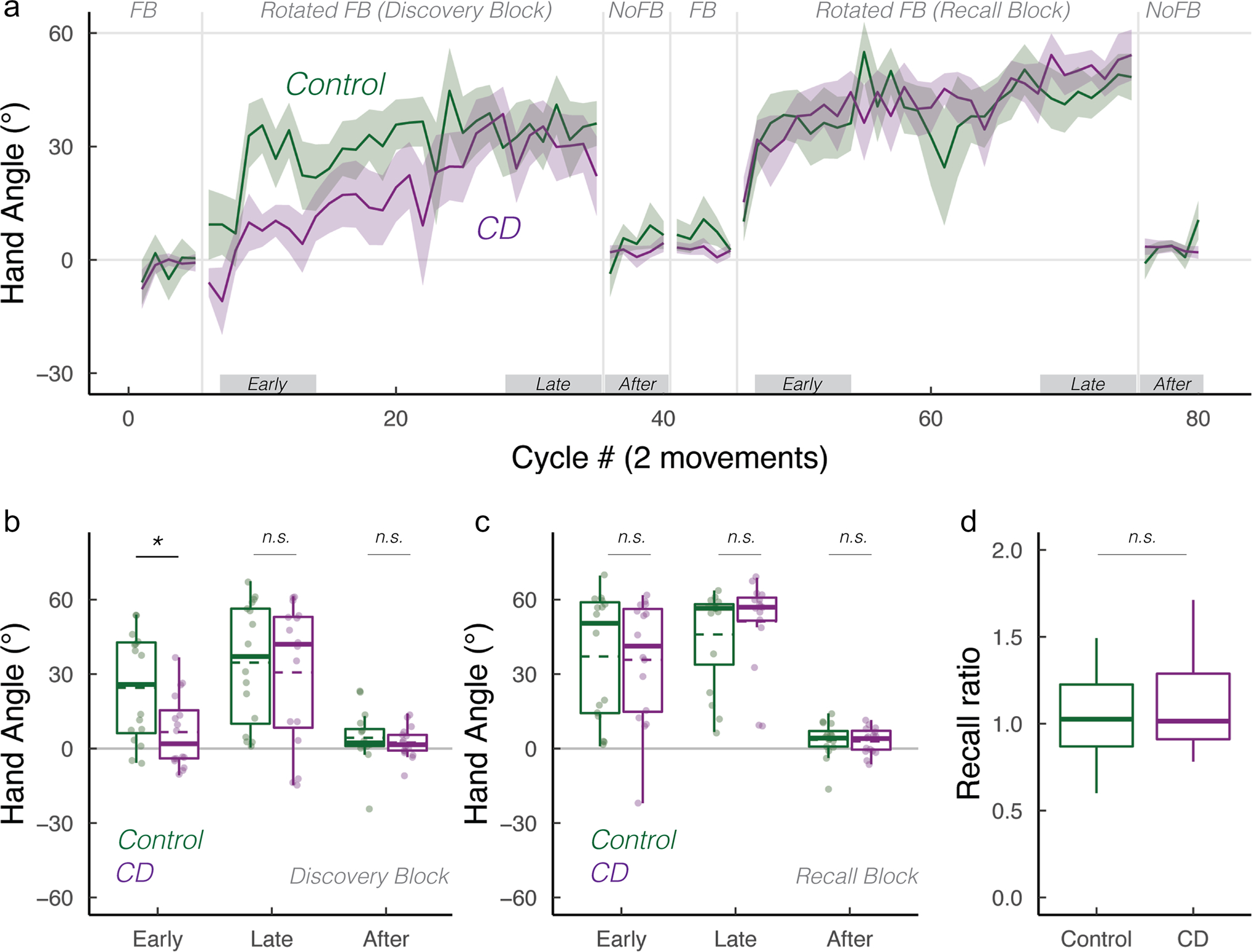
Cerebellar degeneration impairs strategy discovery but not strategy recall. **a** Mean time courses of hand angle for control (dark green) and CD groups (dark magenta). Hand angle is presented relative to the target (0°). **b**, **c** Box plot denotes median hand angles (solid line), mean hand angles (dashed lines), 1st/3rd IQR, and min/max during the three epochs of the **b** Discovery rotation block and **c** Recall rotation block. **d** Recall ratio, quantifying the degree to which late adaptation in the Discovery block was recalled during early adaptation of the Recall block. n.s. denotes that the group comparison between groups was not significant. *Denotes *p* < 0.05. Translucent dots denote individual participants

How does the cerebellum contribute to strategic re-aiming? To examine this, we tested participants with cerebellar degeneration (CD) and age-matched controls (*N* = 16/group) on a visuomotor rotation task designed to require the use of an aiming strategy. After an initial veridical feedback baseline block to familiarize participants with the task environment, the feedback cursor was rotated by 60° (Fig. 1a). To compensate for this rotation, both groups exhibited significant hand angle changes in the opposite direction of the rotation, drawing the cursor closer to the target. When participants were asked to forgo their strategies and re-aim back to the target during the initial no-feedback aftereffect block, both groups were able to “switch-off” their strategies and successfully re-aim back to the target. Both groups exhibited minimal aftereffects, confirming that our delayed feedback manipulation was successful in eliminating implicit recalibration [43, 44]. Upon re-exposure to the rotation in the Recall block, participants were able to recall their aiming strategy, and subsequently switch back to aiming directly to the target in the second no-feedback aftereffect block. In sum, the adaptive changes observed in response to the 60° rotation were the result of strategic re-aiming, rather than implicit recalibration (also see Supplemental Materials).

To statistically evaluate performance, we focused on three a priori-defined epochs: early, late, and aftereffect epochs (Fig. 1b, c). For control participants, hand angles deviated significantly from baseline to nullify the rotation during both early adaptations (24.5° (13.0°, 35.9°); *t*(57) = 4.3, *p* < 0.001,*D*_*z*_ = 1.1) and late adaptation in the Discovery block (34.5° (23.0°, 45.9°); *t*(57) = 1.2, *p* = 0.25,*D*_*z*_ = 1.3). For the CD participants, hand angles did not significantly deviate from baseline during early adaptation (6.6° (− 4.8°, 18.1°); *t*(57) = 1.2, *p* = 0.25,*D*_*z*_ = 0.4), evidence of a deficit in strategic discovery. The CD participants did exhibit significant adaptation during the late epoch of the Discovery block (27.8° (16.4°, 39.3°); *t*(57) = 4.9, *p* < 0.001,*D*_*z*_ = 0.9). There was a main effect of Block for both early (*F*(1, 30) = 5.0, *p* = 0.03,$\eta_{p}^{2}=0.5$) and late epochs (*F*(1, 30) = 4.3, *p* = 0.05,$\eta_{p}^{2}=0.4$), demonstrating that hand angles were greater in the Recall Block compared to Discovery Block.

Turning to our main question, we asked whether CD impacts strategy discovery and/or strategy recall. During early adaptation, there was a main effect of group (*F*(1, 49) = 5.2, *p* = 0.03,$\eta_{p}^{2}=0.06$) and a marginal interaction between the group and Block (*F*(1, 30) = 4.3, *p* = 0.05,$\eta_{p}^{2}=0.1$). As can be seen in Fig. 1b, c, the CD group showed attenuated early adaptation during the Discovery block compared to the control group, but a similar level of adaptation during the Recall block. This inference was confirmed in a post hoc comparison of the two groups for each of the adaptation blocks (Discovery block: (*t*(26) = 2.3, *p* = 0.02,*D* = 1.0; Recall block: (*t*(26) = 0.2, *p* = 1,*D* = 0.1). Interestingly, during late adaptation, we observed neither a main effect of group (*F*(1, 44) = 0.63, *p* = 0.43,$\eta_{p}^{2}=0.0$) nor an interaction between these variables (*F*(1, 30) = 2.1, *p* = 0.16,$\eta_{p}^{2}=0.1$). Thus, by the end of both rotation blocks, the CD and Control groups were equally successful in nullifying the visuomotor perturbation.

We recognize that focusing on hand angle may not be an appropriate measure of recall given that participants varied in terms of their strategy use over the course of the first rotation block. To account for this variation, we performed a second assay by calculating a “recall ratio,” quantifying the extent to which performance during the early adaptation epoch in the Recall block approached that observed in the late adaptation epoch of the Discovery block (i.e., the early recall epoch normalized by late discovery epoch). A recall ratio of 1 signifies complete strategic recall, below 1 signifies partial strategic recall, and above 1 signifies the use of a larger aiming strategy upon re-exposure to the visuomotor rotation. Based on this measure, both groups exhibited strong recall (Fig. 1d): The control group had a recall ratio of 1.3 (IQR: (0.9, 7.1); Wilcoxon test against 0: *V* = 122, *p* = 0.003), and the CD group had a recall ratio of 1.0 (IQR: (0.8, 2.4); *V* = 110, *p* = 0.03), respectively. Critically, the recall ratio did not differ significantly between groups (*W* = 135, *p* = 0.80), providing further evidence that strategic recall was not impaired in CD.

As shown in Fig. 1c, d, there were large individual differences in the use of an aiming strategy. We note that our sample included 5 participants with SCA-3, a variant that is commonly associated with extra-cerebellar symptoms [49–51]. To test whether the impairment in strategy discovery was especially pronounced in this group, we performed a post hoc analysis, comparing the SCA-3 group vs the other CD participants. There was no difference between these two subgroups (Welsh two-sample *t*-test for samples with unequal variances: *t*(5) = 1.8, *p* = 0.10), although we recognize that the inferences to be drawn from this null result are limited by the small sample size.

**Fig. 2:**
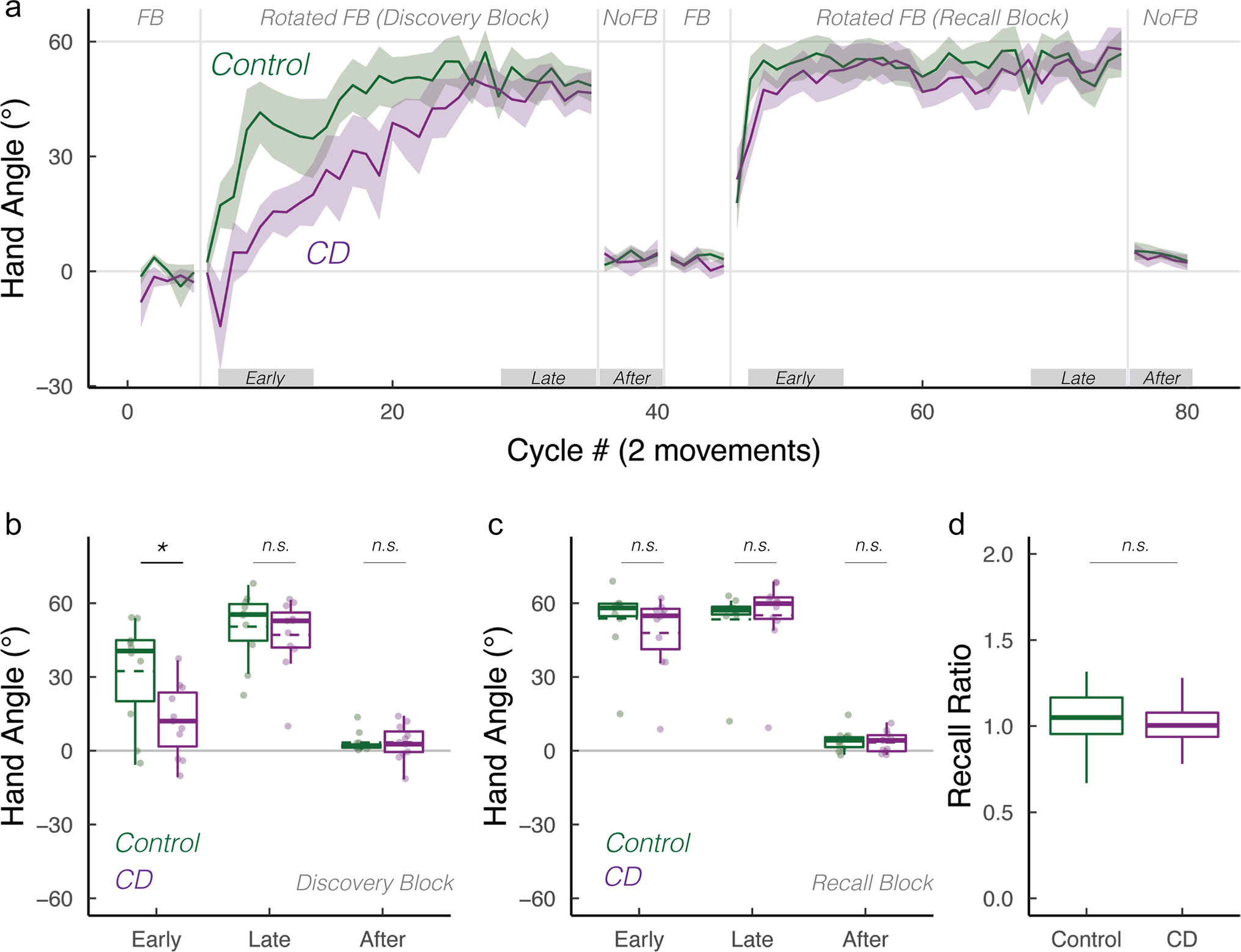
Verification of key findings when analyses were restricted to those showing evidence of strategy use in the Discovery block (“learners”). **a** Mean time courses of hand angle for control (dark green) and CD groups (dark magenta). Hand angle is presented relative to the target (0°). **b**, **c** Box plot denotes median hand angles (solid line), mean hand angles (dashed lines), 1st/3rd IQR, and min/max during the three epochs of the **b** Discovery rotation block and **c** Recall rotation block. **d** Recall ratio, quantifying the degree to which late adaptation in the Discovery block was recalled during early adaptation of the Recall block. n.s. denotes that the group comparison between groups was not significant. *Denotes *p* < 0.05. Translucent dots denote individual participants

Interestingly, the number of participants whose early and late adaptation mean hand angles were close to baseline was similar in the CD and control groups. While this behavior is indicative of an impairment in strategy use, it may reflect a more general performance issue such as inattention to the feedback or failure to fully understand the task instructions. These “non-learners” would also have non-sensical recall ratios since there was no learning to recall. We thus performed a secondary analysis that was limited to “learners,” defined as those participants who had a hand angle significantly greater than zero in the late adaptation epoch of the Discovery block (Fig. 2a, data from 11 controls and 10 CD participants). We repeated our primary analyses with these subgroups, reasoning that group differences here would be a cleaner test of the process of discovery and strategy recall. Our key comparisons were replicated: adaptation in the CD group was selectively impaired in the early epoch of the Discovery block (Fig. 2b; *t*(16) = 2.5, *p* = 0.04,*D* = 1.1). In contrast, the groups did not differ in all other epochs (Fig. 2b, c; Discovery block, late: *t*(19) = 0.9, *p* = 0.8,*D* = 0.4; Recall block, early: *t*(19) = 0.9, *p* = 0.40,*D* = 0.4; late: *t*(19) = 0.1, *p* = 1,*D* = 0.0). The recall ratio also did not differ between groups (Fig. 2d; *W* = 51, *p*= 0.80), altogether re-affirming that CD selectively impairs strategy discovery, but not strategy recall.

## Discussion

While sensorimotor adaptation is often assumed to be automatic and implicit, motor adaptation tasks can also induce volitional and strategic re-aiming [4, 7, 10, 52]. Recent work has indicated that the integrity of the cerebellum is not only essential for implicit recalibration, but is also important for successful strategic re-aiming [35, 36]. Here we set out to ask *how* the cerebellum contributes to strategy use. We compared patients with cerebellar degeneration (CD) and matched controls on a visuomotor adaptation task in which performance changes were dependent on strategic re-aiming, using a design that allowed us to examine strategy discovery and strategy recall. Participants with CD exhibited attenuated early learning upon initial exposure to the visuomotor rotation, evidence of a strategy discovery impairment. However, the CD group eventually reached levels of learning similar to that of controls. Moreover, the CD group was successful in recalling their strategy upon re-exposure to the rotation. Taken together, the results suggest that the cerebellum may play a selective role in strategy discovery but not strategy recall.

Superficially, the impairment in strategy discovery observed in the current study would appear to be at odds with the findings of Wong et al. (2019) who reported that CD participants showed similar performance to control participants on a visuomotor adaptation task in which implicit recalibration was rendered irrelevant [35]. However, there are two key methodological differences between our study and Wong et al. [1] The participants in Wong et al. had direct vision of the hand which was on the same horizontal plane as the cursor and [2] Wong et al. provided visual landmarks on the screen, referents that could be used to support an aiming strategy. These features likely made the task less about *self*-discovery and more about *guided*-discovery of a re-aiming strategy. Having the target, cursor, and hand in the same plane makes it easier to attend to these three inputs to compute the size of the angular rotation and modify a re-aiming strategy to improve performance. In our study, we expect the comparison is less direct, requiring a comparison of an observed cursor and target, short-term memory of a planned direction, and perhaps kinesthetic input from the unattended hand. More importantly, the visual aiming landmarks in Wong et al. provide a very salient cue and were at positions that would lead to successful performance should the participant aim to one of the landmarks. Because they are at fixed, discretized locations, we anticipate they limited the need to explore the entire workspace. By not including the landmarks, our design put the stress on self-discovery. Indeed, the null finding in Wong et al. (2019) is convergent with that of prior work from our lab in which we showed that CD participants can quickly implement a successful re-aiming when visual landmarks are on the screen and participants are instructed to use them [34].

The cerebellum’s role in strategic re-aiming may parallel its role in cognition at large: One recent hypothesis centers on the cerebellum facilitating “mental simulations” [53]. For example, patients with CD exhibit impairments in covertly rotating a visual object held in mind or in covertly “moving” along a mental number line during simple addition. Interestingly, strategy discovery in a visuomotor rotation task is also thought to require mental simulation, that is, the ability to mentally transform a motor plan that is initially oriented toward the target and then rotated to a direction aligned with the hypothesized aiming solution [47, 54–56]. A deficit in mental simulation may have impaired the CD group’s ability to rapidly discover a re-aiming strategy.

What is clear is that degeneration of the cerebellum does not disrupt simple memory retrieval, underscoring that the discovery deficit in the CD group does not arise from some general problem with strategy use or generic problem in task performance. This hypothesis is consistent with previous visuomotor rotation studies that have shown that CD participants do not show deficits in recalling an already discovered strategy or implementing a strategy provided through verbal instructions [34, 35]. Interestingly, their normal performance during recall (and at the end of the Discovery block) would suggest that mental simulation is not required when a strategy is well-established. That is, successful recall in our current design could be achieved by memorizing two aiming locations, one for each target [47].

There are alternative hypotheses to consider when interpreting the discovery deficit in the CD group. For example, the ataxic movements of the CD participants may make it hard to evaluate a strategy since the actual movement may deviate substantially from the intended movement. Related to this idea, the CD participants may be more likely to attribute the observed error to their ataxia rather than assume an external perturbation of the feedback [57–61]. We tested this idea in a previous study [36] by assessing strategic re-aiming in control participants when we added random noise to a rotated cursor on each trial, with the noise distribution set to match that observed in reaching movements of people with CD (also see [62]). We reasoned that the added noise might heighten a “credit assignment” problem (“Did I miss the target because the world is changed or because my motor system is noisy?”) and thus produce an attenuated learning function, similar to what we see in CD. The results did not support this hypothesis. Nonetheless, the idea may still be relevant in terms of the performance of the CD group where noisy movements are chronic, rather than imposed in an “acute” manner in our study with control participants. That is, the CD group may find it harder to determine if a re-aiming strategy was appropriate or needed to be changed due to their ataxia.

The inferences we can draw in terms of brain-behavior relationships are limited by the heterogeneity of our patient sample. Of note, our sample did include a large number of patients with SCA-3, a genetic variant that typically produces symptoms indicative of cerebellar and extra-cerebellar involvement [63] and is associated with depressed cognitive function [49]. We failed to find an association between SCA subtypes and the impairment in strategy discovery, although the sample size is quite small for any subtype analysis. Identifying *which* cerebellar (and extra-cerebellar structures) are critical for strategic discovery is an exciting area for future research. Structural imaging studies (e.g., [20, 22]) can employ larger samples to correlate changes in brain anatomy and re-aiming behavior; functional imaging studies (e.g., [64, 65]) could be used to identify regions within the cerebellum associated with strategy discovery and how these regions interact with the rest of the brain to support this volitional process.

The current results add to the growing evidence that the functional contribution of the cerebellum in adaptation tasks extends beyond its well-established role in the implicit recalibration of the sensorimotor system [66–69]. By using a task that minimizes the contribution of implicit recalibration, we provided clear evidence that CD disrupts strategic re-aiming. Importantly, the observed dissociation between strategy discovery and strategy recall points to how the cerebellum might contribute to strategic re-aiming, with the latter hypothesized to require the simulation of internal [53, 70] and physical states [71, 72]. More generally, postulating constraints on the computational role of the cerebellum will be essential for advancing our understanding of how this subcortical structure interacts with the rest of the brain to support our motor and mental competencies.

## Supplementary Material

2

## Figures and Tables

**Table 1:** CD and matched control participants. SARA scores can range from 0 to 40 (where higher scores indicate greater symptomology). MoCA scores can range from 0 to 30 (where lower scores indicate greater impairment). Mean ± SD is provided

Group	*N*	Age	Sex	Handedness	Years of education	MoCA	SARA

CD	16	57.5 ± 11.8	5 M, 11 F	15R, 1L	16.9 ± 2.7	26.1 ± 2.1	14.3 ± 5.7
Control	16	59.8 ± 11.7	7 M, 9 F	15R, 1L	17.5 ± 2.0	27.7 ± 1.7	–

**Table 2:** Demographics for the individual participants in the group with cerebellar degeneration (CD) and the age-matched Control group. Participant identification, age, sex, handedness, scores for the Montreal Cognitive Assessment (MoCA), and years of education (YOE) are reported for all participants. MoCA scores ≥ 25 indicate no cognitive impairment. Scores for the Scale for Assessment and Rating of Ataxia (SARA) and the subtype of cerebellar degeneration are reported for the CD group. Higher SARA scores indicate more severe cerebellar symptoms and motor deficits

Cerebellar Degeneration (*n* = 16)								Control (*n* = 16)					
ID	Age	Sex	Hand	Subtype	MoCA	YOE	SARA	ID	Age	Sex	Hand	MoCA	YOE

1	59	M	R	SCA3	28	16	5.5	1	71	M	R	24	18
2	51	F	R	SCA2	25	16	12	2	49	M	R	29	20
3	50	M	R	SCA3	26	19	11	3	60	F	R	24	16
4	50	F	R	SCA1	29	20	10.5	4	38	M	L	30	18
5	65	F	L	SCA2	26	16	14.5	5	62	F	R	28	20
6	64	M	R	Sporadic SCA	22	16	12	6	80	M	R	29	14
7	79	F	R	Unknown	28	18	11	7	61	F	R	29	17
8	79	F	R	Unknown	29	12	21.5	8	60	F	R	27	16
9	38	F	R	Unknown	26	12	23.5	9	58	M	R	29	15
10	57	F	R	Unknown	28	14	22.5	10	47	M	R	27	16
11	58	F	R	Unknown	25	17	18	11	69	F	R	28	18
12	44	F	R	SCA3	29	18	4.5	12	44	F	R	28	18
13	54	F	R	Unknown	28	18	11	13	50	F	R	27	22
14	60	M	R	SCA3	25	18	15.5	14	74	F	R	29	18
15	43	F	R	SCA3	28	18	20	15	62	M	R	27	18
16	69	M	R	SCA6	24	22	15.5	16	71	F	R	28	18

## Data Availability

Data will available at https://github.com/xiaotsay2015
