# Supplementary material for: Cerebellar Degeneration Impairs Strategy Discovery but Not Strategy Recall
Source: Cerebellum. Author manuscript; Available in PMC 2024 Feb 16. (PMC10239782; doi:10.1007/s12311-022-01500-6)
Supplement: 2 [file NIHMS1863693-supplement-2.docx]

# Supplemental Materials

**Adaptive changes primarily arise from explicit re-aiming**: We sought to verify that the adaptive changes in the current experiment should be attributed to strategic re-aiming rather than implicit recalibration. To test this, we examined the hand angle data from the two aftereffect blocks. After the Discovery block, aftereffects did not significantly differ from baseline in both groups (Control: 4.3° [-1.5°, 10.1°]; $t\left( 15 \right)=1.6, p=0.10,D=0.4$; CD: 2.4° [-1.0°, 5.8°]; $t\left( 15 \right)=1.5, p=0.20,D=0.4$). After the Recall block, the Control group again exhibited no aftereffects (3.5° [-0.3°, 7.4°]; $t\left( 15 \right)=1.9, p=0.07,D=0.5$). The CD group exhibited a small aftereffect (2.9° [0.1°, 5.7°]; $t\left( 15 \right)=2.2, p=0.04,D=0.6$), a value that is small relative to the aftereffect observed in studies using designs that should engage implicit recalibration (Controls: 15° - 25°; CD: 5° - 15°) (1–7). Aftereffects neither differed between Blocks (main effect of Block: $F\left( 1, 30 \right)=0.1, p=0.75,\eta_{p}^{2}=0.0$) nor Groups (main effect of Group: $F\left( 1, 58 \right)=0.5, p=0.48,\eta_{p}^{2}=0.0$; Group x Block interaction: $F\left( 1, 30 \right)=0.2, p=0.71,\eta_{p}^{2}=0.0$). In summary, these results indicate that the delayed endpoint feedback manipulation successfully minimized the extent of implicit recalibration. By inference, we assume that the adaptive changes arise from strategic re-aiming (8).

**Comparing learners versus non-learners.** We asked whether the learner/non-learner distinction could be predicted by any demographic characteristics. Using a logistic regression, we found that this distinction, across all participants, was not significantly associated with years of education ($\chi^{2}\left( 1 \right)=0.9, p=0.35$), MoCA scores ($\chi^{2}\left( 1 \right)=0.4, p=0.51$), or handedness ($\chi^{2}\left( 1 \right)=3.1, p=0.08$), nor with SARA scores in the CD group ($\chi^{2}\left( 1 \right)=3.4, p=0.06$).

1. Tsay JS, Parvin DE, Ivry RB. Continuous reports of sensed hand position during sensorimotor adaptation. J Neurophysiol. 2020 Oct 1;124(4):1122–30.

2. Tsay JS, Kim HE, Parvin DE, Stover AR, Ivry RB. Individual differences in proprioception predict the extent of implicit sensorimotor adaptation. J Neurophysiol [Internet]. 2021 Mar 3; Available from: http://dx.doi.org/10.1152/jn.00585.2020

3. Kim HE, Morehead R, Parvin DE, Moazzezi R, Ivry RB. Invariant errors reveal limitations in motor correction rather than constraints on error sensitivity. Commun Biol. 2018 Mar 22;1:19.

4. Morehead R, Taylor JA, Parvin DE, Ivry RB. Characteristics of Implicit Sensorimotor Adaptation Revealed by Task-irrelevant Clamped Feedback. J Cogn Neurosci. 2017 Jun;29(6):1061–74.

5. Tseng Y-W, Diedrichsen J, Krakauer JW, Shadmehr R, Bastian AJ. Sensory prediction errors drive cerebellum-dependent adaptation of reaching. J Neurophysiol. 2007 Jul;98(1):54–62.

6. Butcher PA, Ivry RB, Kuo S-H, Rydz D, Krakauer JW, Taylor JA. The cerebellum does more than sensory prediction error-based learning in sensorimotor adaptation tasks. J Neurophysiol. 2017 Sep 1;118(3):1622–36.

7. Martin TA, Keating JG, Goodkin HP, Bastian AJ, Thach WT. Throwing while looking through prisms: I. Focal olivocerebellar lesions impair adaptation [Internet]. Vol. 119, Brain. 1996. p. 1183–98. Available from: http://dx.doi.org/10.1093/brain/119.4.1183

8. Morehead R, Qasim SE, Crossley MJ, Ivry R. Savings upon Re-Aiming in Visuomotor Adaptation. J Neurosci. 2015 Oct 21;35(42):14386–96.
